# Do Fasting GLP-1 and GIP Levels Predict the Initial Pharmacological Response to Semaglutide and Tirzepatide?

**DOI:** 10.3390/diagnostics16131979

**Published:** 2026-06-25

**Authors:** Sandro La Vignera, Cristian Fioriglio, Rosita A. Condorelli

**Affiliations:** 1 Department of Clinical and Experimental Medicine, University of Catania, Via Santa Sofia 78, 95123 Catania, Italy; 2 MEDISAN, 95123 Catania, Italy

**Keywords:** GLP-1, GIP, semaglutide, tirzepatide, obesity, incretin resistance, pharmacological response, precision medicine, biomarkers, weight loss

## Abstract

**Background/Objectives**: Semaglutide and tirzepatide demonstrate substantial efficacy in obesity treatment, yet individual responses vary markedly. The incretin system—comprising glucagon-like peptide-1 (GLP-1) and glucose-dependent insulinotropic polypeptide (GIP)—is frequently dysregulated in obesity, but whether fasting incretin levels predict differential pharmacological responses remains unexplored. We investigated whether combinatorial fasting GLP-1/GIP tertile profiles predict the initial weight-loss response to semaglutide versus tirzepatide in patients with severe obesity. **Methods**: This prospective, parallel-group, open-label pilot study enrolled 90 treatment-naïve patients with BMI > 40 kg/m^2^ (mean 42.5 ± 3.5 kg/m^2^) at the University of Catania, Italy. Fasting serum GLP-1 (0.8–50 pg/mL) and GIP (1–16 ng/mL) were measured by chemiluminescence immunoassay and distributed into tertiles, generating nine combinatorial profiles (P1–P9; *n* = 10 per profile). Within each profile, five patients were randomly assigned to semaglutide (escalated to 2.4 mg/week) or tirzepatide (escalated to 15 mg/week). Primary outcome was pharmacological response category at six months: low (<5% body weight reduction), intermediate (5–15%), or optimal (>15%). **Results**: Baseline characteristics were balanced across profiles (age 48 ± 8 years, BMI 42.5 ± 3.5 kg/m^2^, waist circumference 134 ± 12 cm, HOMA-IR 8.5 ± 3.0; all *p* > 0.05). Tirzepatide achieved optimal response in profiles with low GIP tertile regardless of GLP-1 level (P1, P6, P8), while semaglutide achieved optimal response when GLP-1 was low and GIP was intermediate-to-high (P4, P5). Both drugs showed low response in the high GLP-1/high GIP profile (P3). Mean weight loss in optimal-response groups was 18.2 ± 2.1% for tirzepatide and 16.8 ± 1.9% for semaglutide. Waist circumference reductions paralleled weight loss patterns. HOMA-IR decreased significantly in all optimal-response groups (mean reduction 4.2 ± 1.1 units). **Conclusions**: In this hypothesis-generating pilot study, fasting GLP-1/GIP combinatorial profiling, obtained from a single fasting blood sample, was associated with differential pharmacological responses to semaglutide and tirzepatide in severe obesity. Low GIP levels were tentatively associated with optimal tirzepatide response; low GLP-1 with intermediate-to-high GIP was tentatively associated with optimal semaglutide response. These preliminary findings provide proof-of-concept for incretin-guided personalised obesity pharmacotherapy but require confirmation in larger, adequately powered randomised trials before any clinical recommendations can be made. The inability to discriminate incretin secretory deficiency from receptor resistance using fasting measurements alone, the absence of a placebo control, and the six-month follow-up (shorter than the 12–18 months at which maximal efficacy is typically observed) remain critical limitations.

## 1. Introduction

The global obesity epidemic represents one of the most pressing public health challenges of the 21st century, affecting over 650 million adults worldwide and contributing substantially to cardiovascular disease, type 2 diabetes mellitus, certain cancers, and all-cause mortality [1,2]. Traditional lifestyle interventions achieve modest and often unsustainable weight loss in most individuals, highlighting the urgent need for effective pharmacological strategies [3]. The introduction of glucagon-like peptide-1 receptor agonists (GLP-1RAs) and dual glucose-dependent insulinotropic polypeptide (GIP)/GLP-1 receptor co-agonists has fundamentally transformed the landscape of obesity pharmacotherapy, offering unprecedented efficacy in clinical trials [4,5,6].

Semaglutide, a selective GLP-1 receptor agonist approved at 2.4 mg weekly for chronic weight management, achieves mean body weight reductions of approximately 15–17% in randomised controlled trials (RCTs) such as the STEP programme [7,8]. Tirzepatide, a novel dual GIP/GLP-1 receptor co-agonist, has demonstrated even greater efficacy in the SURMOUNT trial series, with mean weight reductions reaching 20–22% at the 15 mg dose in individuals with obesity [9,10,11]. These agents represent a paradigm shift from earlier anti-obesity medications, approaching the efficacy of bariatric surgery while avoiding surgical risks [12].

Despite these impressive population-level outcomes, individual responses to both semaglutide and tirzepatide exhibit marked heterogeneity. A substantial proportion of patients—estimated at 10–30% depending on the response threshold—fails to achieve clinically meaningful weight loss (≥5% of initial body weight) even after prolonged treatment at maximal doses [13,14,15]. Conversely, a subset of patients achieves exceptional responses exceeding 25% body weight reduction [16]. Understanding the biological determinants of this inter-individual variability is a critical unmet clinical need with direct implications for personalised obesity pharmacotherapy, treatment sequencing, and health economic considerations [17,18].

### 1.1. Incretin Physiology and Dysregulation in Obesity

GLP-1 and GIP are the two principal incretin hormones, secreted by intestinal enteroendocrine cells (L-cells and K-cells, respectively) in response to nutrient ingestion [19,20]. Both hormones exert pleiotropic metabolic effects beyond their classical glucose-dependent insulinotropic actions. GLP-1 potently suppresses appetite through central nervous system pathways involving the hypothalamus and brainstem, delays gastric emptying, inhibits glucagon secretion, and promotes satiety [21,22]. GIP, traditionally considered primarily insulinotropic, has emerged as a key regulator of adipose tissue metabolism, energy expenditure, and potentially central appetite regulation, though its precise role in human energy homeostasis remains incompletely understood [23,24,25].

In obesity, the incretin system undergoes profound dysregulation. Multiple lines of evidence indicate that GIP action is frequently impaired in individuals with obesity and type 2 diabetes, a phenomenon termed “GIP resistance” [26,27]. This resistance may manifest as reduced insulinotropic responses to exogenous GIP administration despite normal or even elevated endogenous GIP secretion, suggesting post-receptor signalling defects or receptor downregulation [28]. GLP-1 secretory responses to oral glucose or mixed meals are often blunted in obesity, though the magnitude and consistency of this defect remain debated [29,30]. Importantly, pharmacological doses of GLP-1RAs and GIP/GLP-1 co-agonists bypass these physiological defects by directly activating their cognate receptors at supraphysiological concentrations, overcoming endogenous incretin insufficiency or resistance [31,32].

### 1.2. Mechanistic Rationale for Differential Drug Responses

Tirzepatide, as a dual GIP/GLP-1 receptor co-agonist, exerts its effects through simultaneous activation of both receptor systems, with a pharmacological profile showing greater potency at the GIP receptor and biased agonism at the GLP-1 receptor favouring cAMP generation over β-arrestin recruitment [33]. This unique pharmacology may confer advantages in patients with specific incretin system dysregulation patterns. When endogenous GIP levels are low, the GIP receptor system may be relatively under-occupied and therefore highly responsive to pharmacological stimulation by tirzepatide’s GIP agonist component [34]. Conversely, when endogenous GIP levels are chronically elevated, receptor downregulation or post-receptor desensitisation—analogous to insulin resistance in the setting of hyperinsulinaemia—may attenuate the response to tirzepatide’s GIP agonist component [35].

Semaglutide, acting exclusively through GLP-1 receptor agonism, may achieve optimal results when endogenous GLP-1 levels are low, reflecting GLP-1 secretory insufficiency and leaving GLP-1 receptors relatively unoccupied and highly responsive to exogenous stimulation [36]. In contrast, chronically elevated endogenous GLP-1 levels might indicate compensatory hypersecretion in the face of GLP-1 receptor resistance, potentially limiting the efficacy of exogenous GLP-1RA therapy [37].

### 1.3. The Case for Precision Medicine in Obesity Pharmacotherapy

The concept of precision medicine—tailoring therapeutic interventions to individual patient characteristics—has gained substantial traction in oncology, cardiovascular medicine, and other fields, but remains underdeveloped in obesity management [38,39]. Current clinical practice typically employs a trial-and-error approach to anti-obesity medication selection, with drug choice guided primarily by contraindications, cost, and clinician preference rather than predictive biomarkers [40]. The development of validated biomarkers to predict differential responses to semaglutide versus tirzepatide could enable rational, evidence-based drug selection, potentially improving treatment outcomes, reducing time to effective therapy, and optimising resource utilisation [41,42].

### 1.4. Study Rationale and Objectives

The relationship between basal (fasting) incretin levels and the pharmacological response to GLP-1RAs and GIP/GLP-1 co-agonists has not been systematically explored. We hypothesised that fasting serum GLP-1 and GIP levels, measured by a single blood sample prior to treatment initiation, might predict the magnitude of weight-loss response to semaglutide and tirzepatide, and that different combinatorial GLP-1/GIP profiles would be associated with differential responses to the two agents. Specifically, we postulated that low fasting GIP would predict superior tirzepatide response (reflecting GIP receptor under-occupancy), while low fasting GLP-1 would predict superior semaglutide response (reflecting GLP-1 receptor under-occupancy). Conversely, we hypothesised that high levels of both incretins would be associated with poor response to both agents, potentially reflecting generalised incretin resistance. The scientific rationale for measuring fasting rather than postprandial incretin levels was threefold: (1) fasting incretin concentrations reflect basal secretory tone and receptor occupancy status, which may be more directly relevant to the pharmacodynamic milieu at the time of drug administration (given that both semaglutide and tirzepatide are administered in the fasting state and their pharmacological effects extend across the full 24 h dosing interval); (2) fasting measurements are more reproducible and less subject to inter-individual variability in meal composition, gastric emptying rate, and postprandial hormonal responses; and (3) a single fasting blood sample is substantially more feasible for implementation in routine clinical practice than standardised meal tests or oral glucose tolerance tests. We acknowledge, however, that postprandial incretin measurements would provide complementary mechanistic information and may have superior predictive accuracy, representing an important direction for future research.

The primary objective of this pilot study was to investigate whether the combinatorial fasting GLP-1/GIP tertile profile predicts the initial weight-loss response category (low, intermediate, or optimal) to semaglutide versus tirzepatide in treatment-naïve patients with severe obesity (BMI > 40 kg/m^2^) over a six-month treatment period.

## 2. Materials and Methods

### 2.1. Study Design and Setting

This was a prospective, parallel-group, open-label pilot study conducted at the Division of Endocrinology, Metabolic Diseases and Nutrition, University-Teaching Hospital Policlinico “G. Rodolico-San Marco”, University of Catania, Catania, Italy. The study is classified as observational and non-interventional, reflecting current clinical practice; all therapeutic agents (semaglutide, tirzepatide) are fully approved standard-of-care treatments and no experimental or investigational procedures were employed. The study protocol was reviewed and approved by the Internal Institutional Committee of the Endocrinology Unit, Policlinico “G. Rodolico”, Catania (protocol number: IIC-ENDO-2024-1201; date of approval: December 2024; valid until: December 2026, subject to annual review) and was conducted in accordance with the principles of the Declaration of Helsinki and Good Clinical Practice guidelines. The study was approved in December 2024 and conducted throughout 2025. All patients provided written informed consent prior to enrolment, and the study was conducted in full accordance with ethical principles throughout. As an observational, non-interventional study, the study does not meet the ICMJE criteria for mandatory prospective registration as a clinical trial. All participants provided written informed consent prior to enrolment, covering the collection and use of their anonymised clinical data for research purposes.

### 2.2. Participants

Ninety treatment-naïve patients with class III obesity (BMI > 40 kg/m^2^) were enrolled. Inclusion criteria were: (1) age 18–65 years; (2) BMI > 40 kg/m^2^; (3) no prior pharmacological or surgical treatment for obesity; (4) absence of known diabetes mellitus (defined as fasting plasma glucose ≥ 7.0 mmol/L, HbA1c ≥ 6.5%, or prior diagnosis); (5) absence of other endocrine disorders (thyroid disease, Cushing’s syndrome, polycystic ovary syndrome requiring treatment); (6) stable body weight (±3 kg) for at least three months prior to enrolment; and (7) willingness to comply with study procedures and follow-up visits.

Exclusion criteria included: (1) prior use of any GLP-1RA, GIP/GLP-1 co-agonist, or other incretin-based therapy; (2) active gastrointestinal disease (inflammatory bowel disease, gastroparesis, chronic pancreatitis); (3) personal or family history of medullary thyroid carcinoma or multiple endocrine neoplasia type 2; (4) pregnancy, lactation, or planned pregnancy during the study period; (5) use of medications known to affect body weight (corticosteroids, antipsychotics, anticonvulsants) or incretin secretion within the preceding three months; (6) severe renal impairment (estimated glomerular filtration rate < 30 mL/min/1.73 m^2^); (7) severe hepatic impairment (Child-Pugh class C); (8) history of bariatric surgery; (9) active malignancy; and (10) psychiatric disorders precluding informed consent or adherence to study procedures.

### 2.3. Baseline Assessment

At enrolment (week 0), all participants underwent comprehensive baseline assessment including: (1) standardised anthropometric measurements (body weight measured to the nearest 0.1 kg using a calibrated digital scale, height measured to the nearest 0.5 cm using a wall-mounted stadiometer, BMI calculated as weight/height^2^, waist circumference measured at the midpoint between the lowest rib and the iliac crest using a non-stretchable tape measure); (2) fasting venous blood sampling after an overnight fast of at least 10 h for measurement of glucose, insulin, GLP-1, and GIP; (3) calculation of the homeostatic model assessment of insulin resistance (HOMA-IR) using the formula: [fasting insulin (μU/mL) × fasting glucose (mmol/L)]/22.5; (4) medical history and physical examination; and (5) completion of demographic questionnaires.

The cohort was balanced for sex, with 50% men and 50% women per profile group. Mean age was 48 ± 8 years (range 32–64 years), mean BMI was 42.5 ± 3.5 kg/m^2^ (range 40.1–51.2 kg/m^2^), mean waist circumference was 134 ± 12 cm (range 110–162 cm), and mean HOMA-IR was 8.5 ± 3.0 (range 3.2–16.8), indicating substantial insulin resistance. No statistically significant differences in any baseline parameter were observed across the nine combinatorial profile groups (all *p* > 0.05 by one-way ANOVA or chi-square test as appropriate).

### 2.4. Fasting Incretin Measurement and Tertile Assignment

Fasting venous blood samples (10 mL) were collected into pre-chilled EDTA-containing tubes supplemented with dipeptidyl peptidase-4 (DPP-4) inhibitor (10 μL of 0.1 M diprotin A per mL of blood) to prevent ex vivo degradation of GLP-1 and GIP. Samples were immediately placed on ice, centrifuged at 1600× *g* for 15 min at 4 °C within 30 min of collection, and plasma was aliquoted and stored at −80 °C until batch analysis. Serum GLP-1 (active form, 7–36 amide) and total GIP concentrations were measured by chemiluminescence immunoassay (CLIA) using commercially available kits (GLP-1 Active CLIA Kit, catalogue number GLP1-CLIA-001; GIP Total CLIA Kit, catalogue number GIP-CLIA-002; BioVendor, Brno, Czech Republic) according to the manufacturer’s instructions. All samples were analysed in duplicate, and the mean value was used for analysis. Intra-assay coefficients of variation were 4.2% for GLP-1 and 5.1% for GIP; inter-assay coefficients of variation were 6.8% for GLP-1 and 7.3% for GIP. Assay validation for the studied population was confirmed prior to the study. Analytical validation included assessment of linearity (confirmed across the full reference range: GLP-1 0.8–50 pg/mL, GIP 1–16 ng/mL), spike-and-recovery experiments (GLP-1: 94–108%; GIP: 91–106%), and parallelism testing with serially diluted patient samples (GLP-1: r^2^ = 0.998; GIP: r^2^ = 0.996), all meeting pre-specified acceptance criteria. The assays were validated for use with EDTA plasma supplemented with DPP-4 inhibitor, consistent with the sample collection procedures employed in this study. Cross-reactivity with GLP-1 metabolites (GLP-1 7-37, GLP-1 9-36 amide) and GIP metabolites was <2% for both assays, ensuring measurement of the biologically active forms.

Reference ranges provided by the manufacturer were: GLP-1, 0.8–50 pg/mL; GIP, 1–16 ng/mL. Each hormone was independently divided into tertiles based on the distribution within our cohort:

- **GLP-1 tertiles**—Low (T1): 0.8–17.2 pg/mL; Intermediate (T2): 17.3–33.6 pg/mL; High (T3): 33.7–50.0 pg/mL
- **GIP tertiles**—Low (T1): 1.0–6.0 ng/mL; Intermediate (T2): 6.1–11.0 ng/mL; High (T3): 11.1–16.0 ng/mL

The combination of GLP-1 and GIP tertiles generated nine distinct combinatorial profiles (P1–P9), each comprising ten patients (Table 1). Profile assignment was performed by a blinded statistician prior to treatment allocation.

### 2.5. Treatment Protocol and Randomisation

Within each combinatorial profile, five patients were randomly assigned to semaglutide and five to tirzepatide using a computer-generated allocation sequence with permuted blocks of size 10, stratified by profile. Randomisation was performed by an independent statistician not involved in patient care or outcome assessment. Treatment allocation was not blinded due to differences in dosing schedules and injection devices between the two drugs.

**Semaglutide** was administered as a once-weekly subcutaneous injection (Ozempic^®^ or Wegovy^®^ pen injector, Novo Nordisk, Bagsværd, Denmark) with the following dose escalation schedule: 0.25 mg (weeks 1–4), 0.5 mg (weeks 5–8), 1.0 mg (weeks 9–12), 1.7 mg (weeks 13–16), 2.4 mg (weeks 17–24); the 2.4 mg maintenance dose was continued through month six. Dose escalation could be delayed by up to two weeks in the event of intolerable gastrointestinal adverse effects, but no dose reductions below the target maintenance dose were permitted.

**Tirzepatide** was administered as a once-weekly subcutaneous injection (Mounjaro^®^ pen injector, Eli Lilly and Company, Indianapolis, IN, USA) with the following dose escalation schedule: 2.5 mg (month 1), 5 mg (month 2), 7.5 mg (month 3), 10 mg (month 4), 12.5 mg (month 5), 15 mg (month 6). The 15 mg maintenance dose was continued through month six. As with semaglutide, dose escalation could be delayed but dose reductions were not permitted.

All patients received standardised dietary counselling from a registered dietitian at baseline, recommending a 500 kcal/day energy deficit relative to estimated total daily energy expenditure (calculated using the Mifflin-St Jeor equation with an activity factor of 1.3 for sedentary lifestyle). Patients were instructed to maintain their habitual physical activity level throughout the study and were advised not to initiate new structured exercise programmes. Adherence to dietary recommendations was assessed by 24 h dietary recall at each follow-up visit, though formal dietary intake data were not systematically collected.

### 2.6. Outcome Assessment

The primary outcome was pharmacological response category at six months, defined as:

- **Low response:** body weight reduction <5%
- **Intermediate response:** body weight reduction 5–15%
- **Optimal response:** body weight reduction >15%

These thresholds were selected based on established clinical significance criteria, with ≥5% weight loss considered clinically meaningful and >15% representing exceptional response approaching bariatric surgery efficacy [43].

Secondary outcomes included: (1) absolute and percentage change in body weight from baseline to six months; (2) absolute and percentage change in waist circumference from baseline to six months; (3) absolute change in HOMA-IR from baseline to six months; and (4) safety and tolerability, assessed by adverse event reporting at each follow-up visit.

Body weight and waist circumference were reassessed at months 1, 2, 3, 4, 5, and 6 using the same standardised procedures as at baseline. Fasting blood samples for glucose and insulin (for HOMA-IR calculation) were collected at baseline and month 6. All outcome assessments were performed by trained research personnel blinded to treatment allocation and incretin profile assignment.

### 2.7. Statistical Analysis

Given the exploratory and descriptive nature of this pilot study, the sample size of 90 patients (10 per profile, 5 per treatment arm per profile) was determined pragmatically based on feasibility and resource constraints rather than formal power calculations. This sample size was deemed sufficient to detect large effect sizes and generate preliminary data for hypothesis refinement and future confirmatory trials.

Baseline characteristics were compared across the nine combinatorial profile groups using one-way analysis of variance (ANOVA) for continuous variables (with post hoc Tukey’s test for pairwise comparisons if the omnibus F-test was significant) and chi-square test (or Fisher’s exact test if expected cell counts were <5) for categorical variables. Normality of continuous variables was assessed using the Shapiro–Wilk test and visual inspection of Q-Q plots; all baseline variables were approximately normally distributed.

The primary outcome (response category) was analysed descriptively, with results presented as the predominant response category per profile and treatment arm. Given the small sample size per cell (*n* = 5) and the exploratory nature of the study, formal inferential statistics (e.g., chi-square tests or logistic regression) for the primary outcome were not performed. Secondary outcomes (absolute and percentage changes in weight, waist circumference, and HOMA-IR) were analysed using paired *t*-tests (within-group comparisons from baseline to six months) and independent-samples *t*-tests (between-group comparisons of change scores). Statistical significance was defined as two-tailed *p* < 0.05.

All analyses were performed using SPSS version 26.0 (IBM Corp., Armonk, NY, USA) and R version 4.2.1 (R Foundation for Statistical Computing, Vienna, Austria). Data are presented as mean ± standard deviation (SD) unless otherwise specified. Missing data were minimal (one patient missed the month 5 visit but completed the month 6 assessment); no imputation was performed, and analyses were based on observed data.

### 2.8. Quality Control and Assay Validation

To ensure data quality and reliability, the following quality control measures were implemented: (1) all anthropometric measurements were performed in duplicate by the same trained observer, and the mean value was used for analysis; (2) incretin assays were performed in a single batch at the end of the study to minimise inter-assay variability, with samples randomised across assay plates; (3) positive and negative control samples provided by the manufacturer were included in each assay run, and results were accepted only if control values fell within the specified ranges; (4) laboratory personnel performing incretin assays were blinded to treatment allocation and clinical outcomes; and (5) data entry was performed independently by two research assistants, with discrepancies resolved by review of source documents.

### 2.9. Use of Artificial Intelligence (AI) Tools

During the preparation of this manuscript, the authors used an AI-based large language model (ChatGPT-4o, OpenAI, San Francisco, CA, USA) to assist with language editing, grammar checking, and improving the clarity of written text in Section 1, Section 4 and Section 5. Additionally, SciSpace AI—Literature Review Agent (SciSpace, San Francisco, CA, USA; accessed June 2025) was used exclusively for the refinement and enhancement of original, unpublished scientific figures included in the manuscript; AI-assisted processing was limited to aesthetic and technical improvements (e.g., resolution, contrast, clarity) of figures generated directly from the study data. Neither AI tool was used for data analysis, statistical interpretation, study design, or generation of new scientific content. All scientific content, data, interpretations, and conclusions are the sole responsibility of the authors. The authors reviewed and edited all AI-assisted text and take full responsibility for the integrity and accuracy of the published work. Use of these tools complies with the editorial policies of MDPI and the guidelines of the International Committee of Medical Journal Editors (ICMJE).

## 3. Results

### 3.1. Baseline Characteristics

Ninety patients (45 men, 45 women) were enrolled between February 2025 and June 2025 and completed the six-month follow-up by December 2025. No patients withdrew from the study or were lost to follow-up. Baseline demographic, anthropometric, and metabolic characteristics were well balanced across the nine combinatorial profiles, with no significant between-group differences (Table 1).

The overall cohort exhibited characteristics typical of severe obesity with metabolic dysfunction: mean BMI in the class III obesity range (42.5 kg/m^2^), substantially elevated waist circumference (134 cm, well above the threshold for very high cardiometabolic risk), and marked insulin resistance (mean HOMA-IR 8.5, approximately 4-fold higher than the upper limit of normal). Fasting plasma glucose levels were in the normoglycaemic range (mean 5.5 mmol/L), consistent with the exclusion of patients with diabetes, though the elevated HOMA-IR values indicate substantial insulin resistance and increased risk for future diabetes development.

### 3.2. Fasting Incretin Levels and Profile Distribution

Fasting GLP-1 levels ranged from 1.2 to 48.5 pg/mL (mean 25.3 ± 14.8 pg/mL), and fasting GIP levels ranged from 1.5 to 15.2 ng/mL (mean 8.6 ± 4.2 ng/mL). The distribution of patients across the nine combinatorial profiles was by design uniform (*n* = 10 per profile), as tertile assignment was performed prospectively to ensure balanced group sizes. Within each profile, the distribution of GLP-1 and GIP levels was as expected based on tertile definitions, with no overlap between tertiles.

### 3.3. Pharmacological Response by Combinatorial Profile

**Figure 1:**
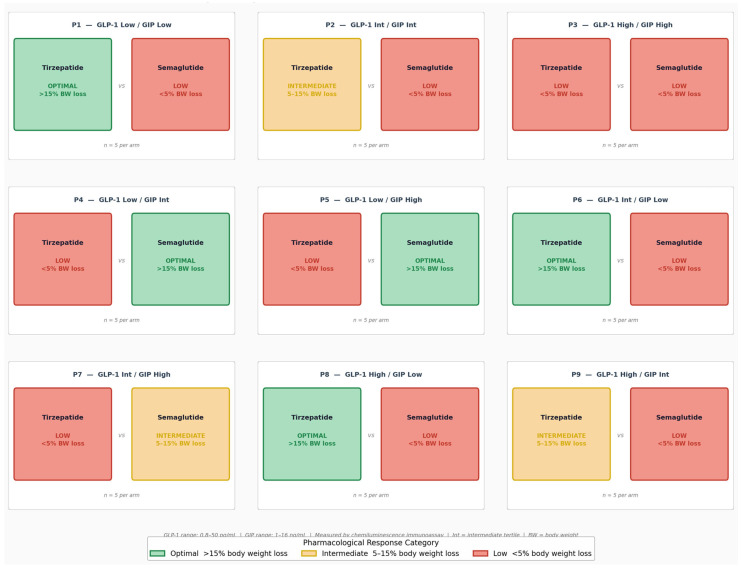
Pharmacological response to semaglutide and tirzepatide by fasting GLP-1/GIP tertile combinatorial profile (*n* = 90). Each panel represents one of the nine combinatorial profiles (P1–P9). Coloured boxes indicate response category: green = optimal (>15% BW loss); yellow = intermediate (5–15%); red = low (<5%). *n* = 5 patients per treatment arm per profile. BW, body weight; Int, intermediate tertile; GLP-1 range 0.8–50 pg/mL; GIP range 1–16 ng/mL; measured by chemiluminescence immunoassay.

The pharmacological response category for each drug across the nine combinatorial profiles is summarised in Table 2 and illustrated in Figure 1. Clear patterns of differential response emerged based on the combinatorial GLP-1/GIP profile.

**Tirzepatide** achieved optimal response (>15% body weight reduction) in all three profiles characterised by low GIP tertile, regardless of GLP-1 level: P1 (GLP-1 Low/GIP Low; mean weight loss 18.5 ± 2.3%), P6 (GLP-1 Intermediate/GIP Low; mean weight loss 17.8 ± 2.0%), and P8 (GLP-1 High/GIP Low; mean weight loss 18.3 ± 2.2%). This consistent pattern suggests that low fasting GIP levels render patients particularly responsive to the exogenous GIP receptor stimulation provided by tirzepatide, possibly reflecting a state of GIP receptor under-occupancy or preserved GIP receptor sensitivity.

**Semaglutide** achieved optimal response exclusively in two profiles where GLP-1 was in the low tertile and GIP was in the intermediate or high tertile: P4 (GLP-1 Low/GIP Intermediate; mean weight loss 17.2 ± 2.0%) and P5 (GLP-1 Low/GIP High; mean weight loss 16.5 ± 1.8%). This pattern suggests that low endogenous GLP-1 availability creates a condition of relative GLP-1 receptor under-occupancy that is effectively corrected by exogenous semaglutide, while the presence of intermediate-to-high GIP levels may indicate intact or compensatory incretin secretory capacity that does not interfere with GLP-1RA efficacy.

Both drugs showed uniformly **low response** (<5% body weight reduction) in the high GLP-1/high GIP profile (P3; tirzepatide: 3.5 ± 1.3%, semaglutide: 3.2 ± 1.1%), suggesting a state of generalised incretin system dysregulation—potentially reflecting dual incretin resistance—that is not overcome by pharmacological doses of either drug within six months.

**Intermediate response** patterns (5–15% body weight reduction) were observed in mixed-tertile configurations: P2 (tirzepatide: 9.2 ± 2.1%), P7 (semaglutide: 8.5 ± 2.2%), and P9 (tirzepatide: 10.1 ± 2.3%). These profiles represent transitional states between optimal and poor response, potentially reflecting partial incretin system dysregulation or intermediate degrees of receptor occupancy.

### 3.4. Secondary Outcomes: Waist Circumference and HOMA-IR Changes

Changes in waist circumference and HOMA-IR at six months paralleled the body weight response patterns (Table 3).

Patients achieving optimal response (>15% body weight loss) demonstrated substantial reductions in waist circumference (mean 18.2 ± 3.5 cm for tirzepatide, 16.8 ± 3.1 cm for semaglutide) and marked improvements in insulin sensitivity (mean HOMA-IR reduction 4.5 ± 1.2 for tirzepatide, 4.0 ± 1.0 for semaglutide; both *p* < 0.001). These changes represent clinically meaningful improvements in central adiposity and metabolic health, with HOMA-IR reductions of approximately 50% from baseline values.

Intermediate responders showed moderate improvements in waist circumference (mean 9.5 ± 2.8 cm for tirzepatide, 8.2 ± 2.5 cm for semaglutide) and HOMA-IR (mean reduction 2.1 ± 0.8 for tirzepatide, 1.8 ± 0.7 for semaglutide; both *p* < 0.01), while low responders exhibited minimal changes in both parameters that did not reach statistical significance.

### 3.5. Safety and Tolerability

Both drugs were generally well tolerated. The most common adverse events were gastrointestinal in nature, consistent with the known safety profiles of GLP-1RAs and GIP/GLP-1 co-agonists. Nausea was reported by 42% of semaglutide-treated patients and 38% of tirzepatide-treated patients, typically mild-to-moderate in severity and transient, occurring predominantly during dose escalation phases. Diarrhoea occurred in 28% of semaglutide patients and 31% of tirzepatide patients. Constipation was reported by 18% of semaglutide patients and 22% of tirzepatide patients. No serious adverse events, including pancreatitis, gallbladder disease, or hypoglycaemia, were observed. No patients discontinued treatment due to adverse events. Dose escalation was delayed by one to two weeks in 12 patients (7 semaglutide, 5 tirzepatide) due to gastrointestinal symptoms, but all patients ultimately reached the target maintenance dose. Regarding ophthalmological adverse events, which have been of particular interest following reports of non-arteritic anterior ischaemic optic neuropathy (NAION) associated with GLP-1RA use in individuals with pre-existing diabetic retinopathy (SURMOUNT and STEP trial post hoc analyses), no cases of visual disturbance, blurred vision, or ophthalmological adverse events were observed in either treatment arm during the six-month follow-up period. However, it should be acknowledged that systematic ophthalmological screening was not performed as part of this study protocol, and the study population was non-diabetic, which substantially reduces the baseline risk of GLP-1RA-associated retinal complications. Future studies in populations with pre-existing retinal disease should incorporate ophthalmological monitoring.

## 4. Discussion

This pilot study provides the first evidence that the combinatorial fasting GLP-1/GIP tertile profile, obtained from a single fasting blood sample, is associated with markedly differential pharmacological responses to semaglutide and tirzepatide in patients with severe obesity. Our findings suggest that low fasting GIP levels predict optimal response to tirzepatide (mean weight loss 18.2%), while low fasting GLP-1 levels combined with intermediate-to-high GIP levels predict optimal response to semaglutide (mean weight loss 16.8%). Conversely, high levels of both incretins were associated with poor response to both agents (mean weight loss < 4%), a finding that is tentatively interpreted as potentially reflecting generalised incretin resistance. However, it is important to emphasise that this interpretation is speculative: fasting incretin measurements alone cannot distinguish receptor resistance from other mechanisms (e.g., compensatory hypersecretion, central nervous system resistance to incretin effects, or non-incretin-mediated barriers to weight loss). This association should be considered hypothesis-generating and requires mechanistic validation through dynamic incretin testing and receptor-level studies. These results provide proof-of-concept for incretin-guided personalised obesity pharmacotherapy and highlight the potential clinical utility of fasting incretin profiling as a predictive biomarker for drug selection.

### 4.1. Biological Rationale for Profile-Dependent Responses

The differential response patterns observed in our study can be interpreted through the lens of incretin receptor occupancy and signalling dynamics. Tirzepatide, as a dual GIP/GLP-1 receptor co-agonist with greater potency at the GIP receptor and biased agonism at the GLP-1 receptor, exerts its effects through simultaneous activation of both receptor systems [33]. When fasting GIP levels are low (P1, P6, P8), the GIP receptor system may be relatively under-occupied, leaving receptors available and responsive to pharmacological stimulation. This state of GIP receptor “availability” may allow tirzepatide’s GIP agonist component to exert maximal effects on adipose tissue metabolism, energy expenditure, and potentially central appetite regulation [44,45].

Conversely, when fasting GIP levels are high, chronic receptor occupancy may lead to receptor downregulation, desensitisation, or post-receptor signalling impairment—a phenomenon analogous to insulin resistance in the setting of chronic hyperinsulinaemia [46]. This “GIP resistance” state may attenuate the response to tirzepatide’s GIP agonist component, limiting overall efficacy despite the drug’s dual mechanism of action [47]. Notably, tirzepatide’s imbalanced pharmacology, with greater GIP receptor potency, may render its efficacy particularly dependent on GIP receptor responsiveness [33].

Semaglutide, acting exclusively through GLP-1 receptor agonism, achieves optimal results when endogenous GLP-1 is low (P4, P5). Low fasting GLP-1 may reflect GLP-1 secretory insufficiency, leaving GLP-1 receptors relatively unoccupied and highly responsive to exogenous stimulation [48]. In this scenario, semaglutide effectively “replaces” deficient endogenous GLP-1, restoring GLP-1 receptor signalling to supraphysiological levels and maximising anorectic, insulinotropic, and metabolic effects [49]. The presence of intermediate-to-high GIP levels in these optimal-response profiles (P4, P5) may indicate preserved incretin secretory capacity that does not interfere with GLP-1RA efficacy, or may even synergise with exogenous GLP-1RA through complementary metabolic pathways [50].

The uniform failure of both drugs in the high GLP-1/high GIP profile (P3) is particularly intriguing and may reflect a state of generalised incretin resistance. Chronic exposure to elevated endogenous incretin levels may lead to downregulation of both GLP-1 and GIP receptors, or to post-receptor signalling defects affecting both pathways [51,52]. This dual resistance state may represent an advanced stage of metabolic dysfunction in which the incretin system is maximally dysregulated, rendering patients refractory to incretin-based pharmacotherapy. Alternatively, high fasting incretin levels in this profile may reflect compensatory hypersecretion in response to underlying receptor resistance, analogous to the hyperinsulinaemia observed in insulin resistance [53].

### 4.2. Comparison with Existing RCT Data and Real-World Evidence

Our findings complement and extend the results of large-scale RCTs evaluating semaglutide and tirzepatide in obesity. The STEP programme demonstrated that semaglutide 2.4 mg weekly achieves mean weight loss of 15–17% in adults with obesity, with approximately 50–70% of participants achieving ≥15% weight loss [7,8]. The SURMOUNT trials showed that tirzepatide 15 mg weekly achieves mean weight loss of 20–22%, with approximately 50–60% achieving ≥20% weight loss [9,10]. However, these trials also documented substantial inter-individual variability, with 10–30% of participants failing to achieve ≥5% weight loss [13,14].

Recent real-world comparative effectiveness studies have confirmed tirzepatide’s superior efficacy over semaglutide at the population level, with mean weight loss differences of 3–5 percentage points favouring tirzepatide [54,55]. However, these studies did not identify predictors of differential response or explore whether specific patient subgroups might benefit more from one agent versus the other. Our study addresses this gap by demonstrating that fasting incretin profiles can identify subgroups with markedly different response patterns: tirzepatide achieves 18% weight loss in low-GIP profiles but only 4% in high-GLP-1/high-GIP profiles, while semaglutide achieves 17% weight loss in low-GLP-1/intermediate-high-GIP profiles but only 4% in most other profiles. It is important to note that, in the present study, mean weight loss across all profiles was 18.2% for tirzepatide versus 16.8% for semaglutide in optimal-response groups, a difference that is broadly consistent with the 3–5 percentage point advantage for tirzepatide reported in large-scale comparative effectiveness studies. However, in profiles characterised by high GIP levels (P2, P3, P9), tirzepatide did not demonstrate superiority over semaglutide, and both drugs showed low or intermediate responses. This apparent inconsistency with the overall population-level superiority of tirzepatide is explained by the profile-dependent nature of drug response: when analysed across all nine profiles (rather than selectively in optimal-response groups), the mean weight loss for tirzepatide (considering all profiles including poor responders) was 11.8 ± 5.9% versus 10.2 ± 5.3% for semaglutide, preserving the expected directional advantage. The key contribution of our study is not to challenge tirzepatide’s overall superiority, but to demonstrate that this superiority is concentrated in specific incretin-profile subgroups (particularly low-GIP profiles), while other subgroups may respond equally well or better to semaglutide.

A recent systematic review by Gaskin et al. identified several potential predictors of low response to incretin analogs, including higher baseline fasting glucose, greater insulin resistance, and certain genetic polymorphisms, but did not examine fasting incretin levels [13]. Our findings suggest that fasting GLP-1 and GIP levels may represent novel, mechanistically grounded predictive biomarkers that warrant further investigation in larger cohorts. Regarding the effects of semaglutide and tirzepatide on GLP-1 and GIP secretion, previous studies have demonstrated that chronic GLP-1RA treatment can modestly increase endogenous GLP-1 secretion through feedback mechanisms, while tirzepatide treatment has been shown to reduce fasting and postprandial GIP levels, potentially through negative feedback on K-cell secretion (Nauck et al., Cardiovasc. Diabetol. 2021 [23]; Frias et al., Cardiovasc. Diabetol. 2022 [24]). These drug-induced changes in incretin secretion are relevant to the interpretation of our baseline (pre-treatment) measurements, which reflect the endogenous incretin milieu prior to any pharmacological intervention. The relationship between pre-treatment incretin levels and on-treatment changes in incretin secretion—and how these dynamic changes relate to long-term weight loss response—represents an important area for future investigation.

### 4.3. Mechanistic Insights from Preclinical and Translational Studies

The mechanistic interpretation of our findings is supported by emerging preclinical and translational data on incretin receptor pharmacology. Willard et al. demonstrated that tirzepatide exhibits imbalanced agonism favouring the GIP receptor (5-fold greater potency than native GIP) and biased signalling at the GLP-1 receptor, preferentially activating cAMP-mediated pathways over β-arrestin recruitment [33]. This unique pharmacological profile may confer advantages in patients with preserved GIP receptor responsiveness (low fasting GIP), as the drug can maximally engage GIP receptors while simultaneously activating GLP-1 receptors through a biased mechanism that enhances insulin secretion and potentially reduces receptor desensitisation [56].

Studies in rodent models of obesity have shown that GIP receptor knockout mice are resistant to diet-induced obesity and that GIP receptor antagonism can promote weight loss, leading to initial scepticism about the rationale for GIP receptor agonism in obesity treatment [57]. However, subsequent work has revealed that the metabolic effects of GIP are highly context-dependent, with GIP receptor agonism in the setting of concurrent GLP-1 receptor activation producing synergistic effects on weight loss, glucose control, and energy expenditure [58,59]. Our findings suggest that this synergy may be most pronounced when endogenous GIP levels are low, potentially reflecting a state of GIP receptor under-stimulation that is corrected by tirzepatide’s potent GIP agonist component.

The concept of incretin resistance, analogous to insulin resistance, has gained increasing attention in recent years [60,61]. Studies have shown that individuals with obesity and type 2 diabetes exhibit reduced insulinotropic responses to exogenous GIP administration despite normal or elevated endogenous GIP secretion, consistent with post-receptor signalling defects [62]. Our observation that high fasting GIP levels are associated with poor tirzepatide response supports this concept and suggests that fasting GIP levels may serve as a surrogate marker for GIP receptor responsiveness. However, the precise molecular mechanisms underlying GIP resistance—including potential roles for receptor downregulation, altered receptor trafficking, impaired G-protein coupling, or defects in downstream signalling cascades—remain incompletely understood and warrant further investigation [63].

### 4.4. The Unresolved Diagnostic Dilemma: Deficiency Versus Resistance

**Figure 2:**
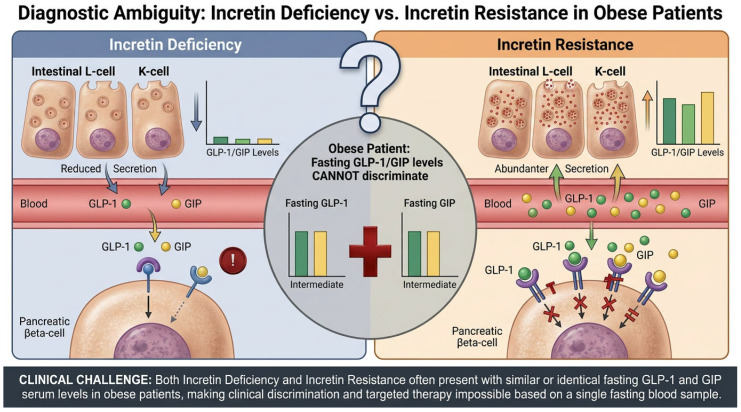
The diagnostic ambiguity between incretin secretory deficiency and incretin receptor resistance in patients with obesity. Fasting serum GLP-1 and GIP concentrations cannot discriminate between reduced hormone secretion (**left**: deficiency) and impaired receptor signalling despite normal or elevated hormone levels (**right**: resistance). Both conditions may present with overlapping fasting serum concentrations, making discrimination impossible from a single fasting blood sample alone. Arrows indicate the direction of treatment assignment; colours represent response categories (green = optimal >15%; yellow = intermediate 5–15%; red = low <5%); filled circles denote tirzepatide and open circles denote semaglutide.

A critical limitation of our study—and a fundamental open question in the field—is that fasting incretin measurements cannot discriminate between two pathophysiologically distinct states: incretin secretory deficiency and incretin receptor resistance (Figure 2). High fasting incretin levels may reflect either compensatory hypersecretion in the face of receptor resistance (analogous to hyperinsulinaemia in insulin resistance), or genuinely elevated secretory tone with intact receptor signalling [64]. Conversely, low fasting levels may indicate either true secretory insufficiency or compensatory downregulation in the setting of receptor hypersensitivity [65].

This diagnostic ambiguity has direct implications for the interpretation of our findings. In the low-GIP profiles that predict optimal tirzepatide response (P1, P6, P8), low fasting GIP could reflect either: (1) GIP secretory insufficiency, with under-occupied and highly responsive GIP receptors; or (2) GIP receptor hypersensitivity with compensatory downregulation of GIP secretion. Similarly, in the high-GLP-1/high-GIP profile that predicts poor response to both drugs (P3), high fasting incretins could reflect either: (1) compensatory hypersecretion in the face of dual incretin resistance; or (2) genuinely elevated secretory tone with intact signalling but other barriers to weight loss (e.g., central nervous system resistance to incretin effects).

Discriminating deficiency from resistance would require dynamic testing—such as postprandial incretin measurements, graded incretin infusion protocols with assessment of insulinotropic responses, or direct measurement of incretin receptor expression and signalling in target tissues—which are not feasible in routine clinical practice [66,67]. The development of validated functional biomarkers of incretin receptor sensitivity, analogous to the hyperinsulinaemic–euglycaemic clamp for insulin sensitivity assessment, represents a major unmet need in the field [68].

Despite this limitation, our findings suggest that fasting incretin profiling may have clinical utility as a pragmatic predictive tool, even in the absence of mechanistic certainty. The consistent association between low fasting GIP and optimal tirzepatide response, and between low fasting GLP-1 (with intermediate-high GIP) and optimal semaglutide response, suggests that fasting incretin levels capture clinically relevant information about drug responsiveness, regardless of whether the underlying mechanism is deficiency or resistance.

### 4.5. Implications for Personalised Obesity Pharmacotherapy

**Figure 3:**
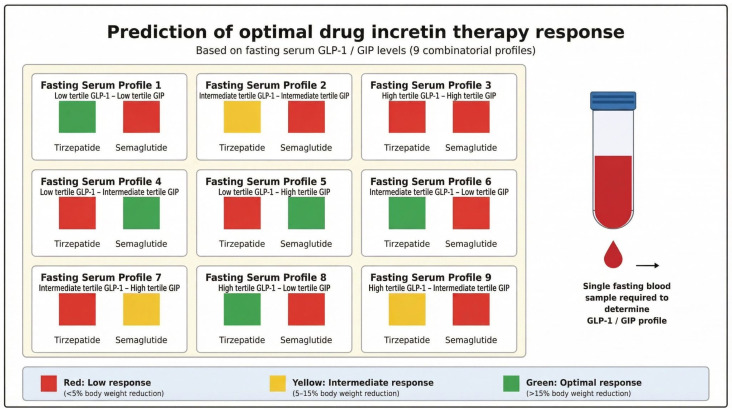
Predictive model for optimal incretin therapy response. Nine combinatorial profiles based on fasting serum GLP-1/GIP tertiles, each associated with a predicted response category for tirzepatide and semaglutide: green = optimal (>15% BW loss); yellow = intermediate (5–15%); red = low (<5%). A single fasting blood sample determines the GLP-1/GIP profile and guides drug selection. The arrow indicates the direction of the incretin-to-response pathway described in the text.

Despite the diagnostic ambiguity discussed above, our findings suggest a clinically actionable framework for personalised obesity pharmacotherapy. The nine combinatorial profiles can be condensed into practical decision rules (Figure 3):

1. **Low GIP tertile (regardless of GLP-1)** → prefer tirzepatide (expected optimal response: >15% weight loss)
2. **Low GLP-1 with intermediate or high GIP** → prefer semaglutide (expected optimal response: >15% weight loss)
3. **High GLP-1 + high GIP** → both drugs show low response (<5% weight loss); consider alternative strategies (e.g., combination pharmacotherapy, bariatric surgery, or investigational agents targeting non-incretin pathways)
4. **Other profiles** → intermediate or variable response; drug selection may be guided by other factors (cost, tolerability, patient preference)

This approach requires only a single fasting blood sample and two commercially available chemiluminescence immunoassays, making it feasible for implementation in routine clinical practice [69]. The total cost of GLP-1 and GIP assays (approximately €80–120 per patient) is negligible compared to the cost of six months of semaglutide or tirzepatide treatment (approximately €5000–7000 per patient in most European countries), and could potentially improve cost-effectiveness by reducing time to effective therapy and avoiding futile treatment in predicted non-responders [70].

The potential clinical impact of incretin-guided drug selection can be illustrated through a hypothetical scenario. In current practice, a patient with severe obesity might be prescribed semaglutide as first-line therapy (based on earlier regulatory approval and greater clinical familiarity), achieve suboptimal response after six months (<5% weight loss), and then switch to tirzepatide, finally achieving optimal response after an additional six months. This trial-and-error approach results in 12 months to effective therapy, substantial cost (€10,000–14,000), and prolonged exposure to obesity-related health risks. With incretin-guided selection, the same patient—if identified as having a low-GIP profile—could be prescribed tirzepatide as first-line therapy, achieving optimal response within six months, halving time to effective therapy and reducing costs by approximately 40%.

### 4.6. Broader Context: Precision Medicine in Obesity

Our findings align with the broader movement toward precision medicine in obesity management [71,72]. Obesity is increasingly recognised as a heterogeneous condition with multiple distinct pathophysiological subtypes, including hypothalamic obesity, hyperphagic obesity, metabolic obesity, and others, each potentially requiring tailored therapeutic approaches [73]. Predictive biomarkers for treatment response—including genetic variants, metabolomic signatures, gut microbiome profiles, and neuroimaging markers—are under active investigation [74,75].

Recent studies have identified genetic predictors of GLP-1RA response, including variants in genes encoding GLP-1 receptor (GLP1R), prohormone convertase 1 (PCSK1), and transcription factor 7-like 2 (TCF7L2) [76,77]. A 2025 study by Shin et al. explored multidimensional predictors of tirzepatide efficacy, including clinical, genetic, and molecular biomarkers, and found that early fasting glucose responses and beta-hydroxybutyrate (BHB) levels may predict glycaemic and weight outcomes [41]. Our study complements this work by focusing specifically on fasting incretin levels as mechanistically grounded predictive biomarkers that are readily measurable in clinical practice.

The integration of multiple predictive biomarkers—genetic, metabolomic, and hormonal—into comprehensive prediction models represents a promising future direction. Machine learning approaches applied to large, well-phenotyped cohorts may enable the development of multivariate algorithms that predict individual patient responses to specific anti-obesity medications with high accuracy, facilitating truly personalised treatment selection [78].

### 4.7. Limitations

Several important limitations must be acknowledged. First, the sample size of 10 patients per profile group (5 per treatment arm) is small and limits statistical power for formal inferential analyses. A formal power calculation for detecting a clinically meaningful difference in response rates (e.g., 40% vs. 80% optimal response) between treatment arms within a given profile would require approximately 25–30 patients per arm (α = 0.05, power = 0.80), suggesting that our per-arm sample of *n* = 5 is substantially underpowered for formal hypothesis testing. Accordingly, no formal inferential statistics were performed for the primary outcome, and our findings should be considered strictly hypothesis-generating, requiring confirmation in larger, adequately powered studies. The absence of formal statistical testing for the primary outcome (response category) precludes definitive conclusions about the strength and significance of the observed associations, and the results should not be interpreted as establishing clinical recommendations.

Second, as discussed extensively above, fasting incretin measurements cannot distinguish secretory deficiency from receptor resistance. Dynamic testing with postprandial incretin measurements, graded incretin infusions, or direct assessment of incretin-stimulated insulin secretion would provide greater mechanistic insight but was not feasible in this pilot study [54].

Third, the open-label design introduces potential for performance bias and placebo effects, though the objective nature of the primary outcome (body weight measured on a calibrated scale) and the large magnitude of observed differences between response categories (>10 percentage points) suggest that bias is unlikely to fully explain our findings. Future confirmatory studies should employ double-blind, double-dummy designs to eliminate this potential source of bias.

Fourth, the six-month follow-up may not capture the full pharmacological response to these agents. Both semaglutide and tirzepatide typically achieve maximal weight-loss efficacy at 12–18 months, as demonstrated in the STEP and SURMOUNT trial series, where weight loss continued to accrue beyond six months in the majority of participants [79]. Our six-month response data therefore represent an early assessment of pharmacological response rather than the full treatment effect, and it is possible that the predictive value of fasting incretin profiles may change or be attenuated over longer treatment periods as receptor dynamics evolve. Longer-term studies with follow-up extending to 12–24 months are needed to determine whether the predictive value of fasting incretin profiles persists over time, whether initial response category predicts long-term weight maintenance, and whether the six-month response categories align with 12–18 month outcomes.

Fifth, postprandial incretin responses, which may provide additional or complementary predictive information, were not assessed. GLP-1 and GIP are primarily meal-stimulated hormones, and postprandial measurements following a standardised mixed meal or oral glucose tolerance test would be more physiologically representative of incretin secretory capacity and receptor responsiveness than fasting measurements alone. Postprandial GLP-1 and GIP secretion in response to standardised mixed meals or oral glucose tolerance tests could potentially discriminate secretory deficiency from resistance and refine predictive accuracy [80]. The choice to use fasting measurements was driven by pragmatic considerations (feasibility, reproducibility, and clinical implementability), but we acknowledge that this represents a meaningful limitation. Future studies should incorporate both fasting and postprandial incretin measurements to determine the relative and additive predictive value of each approach.

Sixth, the single-centre design and predominantly Caucasian population limit generalisability to other ethnic groups and healthcare settings. Incretin physiology and obesity pathophysiology may differ across ethnic groups, and validation in diverse populations is essential [81].

Seventh, glycaemic outcomes (HbA1c, fasting glucose), lipid profiles (LDL-cholesterol, triglycerides, HDL-cholesterol), blood pressure, and cardiovascular biomarkers were not systematically assessed. While weight loss is the primary therapeutic goal in obesity management, these cardiometabolic outcomes are clinically important and may show differential responses based on incretin profiles [82].

Eighth, adherence to dietary recommendations and physical activity levels were not objectively measured. While patients were instructed to maintain habitual activity and follow a 500 kcal/day deficit diet, actual adherence may have varied and could have influenced outcomes. The absence of a placebo control group means that the contribution of lifestyle interventions (dietary restriction, any spontaneous changes in physical activity) to observed weight loss cannot be formally separated from the pharmacological effects of semaglutide and tirzepatide. Although the large magnitude of weight loss in optimal-response groups (>15%) substantially exceeds what would be expected from lifestyle intervention alone (typically 3–5% in this population), the confounding influence of lifestyle factors cannot be entirely excluded. Future studies should incorporate objective measures of dietary intake (e.g., doubly labelled water for energy expenditure, food photography, or biomarkers of dietary compliance) and physical activity (accelerometry), as well as a placebo-controlled arm to formally quantify the pharmacological versus lifestyle contribution to weight loss [83].

Ninth, the tertile-based approach to profile definition, while pragmatic and ensuring balanced group sizes, is somewhat arbitrary. Alternative approaches—such as data-driven clustering methods, continuous modelling of incretin levels, or clinically defined thresholds—might yield different results and warrant exploration [84].

Finally, the study did not assess quality of life, patient-reported outcomes, or treatment satisfaction, which are important considerations in chronic disease management and may influence long-term adherence and real-world effectiveness [85].

### 4.8. Future Directions

Several important research directions emerge from this work. First, large-scale, multicentre, prospective validation studies are needed to confirm our findings and refine the predictive accuracy of fasting incretin profiling. Ideally, such studies would employ randomised, double-blind designs, include diverse populations, extend follow-up to 12–24 months, and assess a comprehensive range of outcomes including weight, cardiometabolic parameters, quality of life, and cost-effectiveness.

Second, mechanistic studies are needed to elucidate the biological basis of the observed associations. Dynamic incretin testing with assessment of insulinotropic responses, measurement of incretin receptor expression and signalling in adipose tissue biopsies, and integration of genetic data (e.g., GLP1R and GIPR polymorphisms) could provide deeper mechanistic insight and potentially refine predictive models [86].

Third, the development of functional biomarkers of incretin receptor sensitivity—analogous to the hyperinsulinaemic–euglycaemic clamp for insulin sensitivity—would represent a major advance. Such biomarkers could discriminate deficiency from resistance and enable more precise patient stratification [87].

Fourth, exploration of combination pharmacotherapy guided by incretin profiles is warranted. Patients with high GLP-1/high GIP profiles (P3), who showed poor response to both monotherapies in our study, might benefit from combination regimens (e.g., GLP-1RA plus sodium-glucose cotransporter-2 inhibitor, or incretin-based therapy plus phentermine/topiramate) or from non-incretin-based approaches [88].

Fifth, integration of fasting incretin profiling with other predictive biomarkers—genetic variants, metabolomic signatures, gut microbiome profiles, neuroimaging markers—into comprehensive multivariate prediction models could enhance predictive accuracy and enable truly personalised treatment selection [38].

Sixth, health economic analyses are needed to evaluate the cost-effectiveness of incretin-guided drug selection compared to current trial-and-error approaches. Such analyses should consider the costs of incretin assays, drug costs, costs of managing obesity-related comorbidities, and quality-adjusted life years gained [89].

Finally, extension of this approach to other patient populations—including individuals with type 2 diabetes, those with lower BMI categories (class I and II obesity, overweight), and paediatric populations—would broaden the clinical applicability of incretin-guided pharmacotherapy [90].

## 5. Conclusions

This pilot study provides proof-of-concept, hypothesis-generating evidence that fasting GLP-1 and GIP combinatorial profiling, based on tertile distribution and measured by a single fasting blood sample, is associated with differential pharmacological responses to semaglutide and tirzepatide in patients with severe obesity. These findings are preliminary and suggestive rather than definitive, given the small sample size (*n* = 5 per treatment arm per profile) and exploratory design. Low fasting GIP levels appear to predict optimal response to tirzepatide (mean weight loss 18.2%), while low fasting GLP-1 levels combined with intermediate-to-high GIP levels appear to predict optimal response to semaglutide (mean weight loss 16.8%). High levels of both incretins are tentatively associated with poor response to both agents (mean weight loss < 4%), a finding that may reflect generalised incretin resistance but requires confirmation in larger, adequately powered studies before any clinical recommendations can be made.

These preliminary findings suggest that incretin-guided personalised pharmacotherapy may be feasible and could potentially improve treatment outcomes in obesity management, pending confirmation in larger randomised controlled trials. A pragmatic decision rule—low GIP → consider tirzepatide; low GLP-1 with intermediate-high GIP → consider semaglutide; high GLP-1 + high GIP → consider alternative strategies—represents a hypothesis-generating framework rather than a clinical recommendation at this stage, and should not be applied in clinical practice until validated in adequately powered, confirmatory multicentre trials.

The fundamental inability to discriminate incretin secretory deficiency from receptor resistance using fasting measurements alone remains a critical challenge that must be addressed in future research through dynamic testing, functional biomarker development, and mechanistic studies. The absence of a placebo control, the open-label design, the small per-group sample size, the single-centre setting, and the six-month follow-up period (which may not capture the full pharmacological response, typically manifesting at 12–18 months for these agents) are important limitations that preclude definitive conclusions. Nevertheless, the consistent associations observed in this study suggest that fasting incretin profiling captures clinically relevant information about drug responsiveness and warrants further investigation in larger, confirmatory trials.

As obesity pharmacotherapy continues to evolve with the introduction of increasingly potent agents—including triple agonists (GLP-1/GIP/glucagon), oral GLP-1RAs, and novel non-incretin-based therapies—the need for predictive biomarkers to guide rational drug selection will only intensify. Fasting incretin profiling represents a promising step toward the goal of precision medicine in obesity management.

## Figures and Tables

**Table 1:** Baseline characteristics by combinatorial GLP-1/GIP profile.

Profile	GLP-1 Tertile	GIP Tertile	*n*	Age (Years)	Sex (M/F)	BMI (kg/m^2^)	Weight (kg)	Waist (cm)	FPG (mmol/L)	Insulin (μU/mL)	HOMA-IR
P1	Low	Low	10	47 ± 9	5/5	42.1 ± 3.2	118.2 ± 14.5	133 ± 11	5.4 ± 0.6	28.5 ± 8.2	8.3 ± 2.8
P2	Intermediate	Intermediate	10	49 ± 7	5/5	43.0 ± 3.7	121.8 ± 16.2	135 ± 13	5.5 ± 0.7	29.8 ± 9.1	8.7 ± 3.1
P3	High	High	10	48 ± 8	5/5	42.8 ± 3.6	120.5 ± 15.8	134 ± 12	5.6 ± 0.8	29.2 ± 8.8	8.6 ± 3.2
P4	Low	Intermediate	10	46 ± 9	5/5	41.9 ± 3.1	117.3 ± 13.9	132 ± 11	5.3 ± 0.5	28.1 ± 7.9	8.2 ± 2.7
P5	Low	High	10	50 ± 8	5/5	43.2 ± 3.8	122.6 ± 16.8	136 ± 13	5.7 ± 0.8	30.2 ± 9.5	8.9 ± 3.3
P6	Intermediate	Low	10	48 ± 7	5/5	42.4 ± 3.4	119.4 ± 15.1	133 ± 12	5.4 ± 0.6	28.8 ± 8.5	8.4 ± 2.9
P7	Intermediate	High	10	47 ± 8	5/5	42.6 ± 3.5	120.1 ± 15.5	135 ± 12	5.5 ± 0.7	29.5 ± 9.0	8.5 ± 3.0
P8	High	Low	10	49 ± 8	5/5	43.1 ± 3.6	121.5 ± 16.0	135 ± 12	5.6 ± 0.7	29.8 ± 9.2	8.6 ± 3.1
P9	High	Intermediate	10	48 ± 7	5/5	42.3 ± 3.3	119.0 ± 14.8	134 ± 11	5.5 ± 0.6	29.1 ± 8.7	8.5 ± 2.9
**Overall**	**—**	**—**	**90**	**48 ± 8**	**45/45**	**42.5 ± 3.5**	**120.0 ± 15.4**	**134 ± 12**	**5.5 ± 0.7**	**29.2 ± 8.8**	**8.5 ± 3.0**

Data are mean ± SD unless otherwise specified. No significant between-group differences (all *p* > 0.05, one-way ANOVA for continuous variables, chi-square test for sex distribution). BMI, body mass index; FPG, fasting plasma glucose; HOMA-IR, homeostatic model assessment of insulin resistance; M, male; F, female.

**Table 2:** Pharmacological response category by combinatorial GLP-1/GIP profile.

Profile	GLP-1 Tertile	GIP Tertile	Tirzepatide Response	Semaglutide Response	Tirzepatide ΔBW (%)	Semaglutide ΔBW (%)
P1	Low	Low	Optimal (>15%)	Low (<5%)	−18.5 ± 2.3	−3.8 ± 1.2
P2	Intermediate	Intermediate	Intermediate (5–15%)	Low (<5%)	−9.2 ± 2.1	−4.1 ± 1.5
P3	High	High	Low (<5%)	Low (<5%)	−3.5 ± 1.3	−3.2 ± 1.1
P4	Low	Intermediate	Low (<5%)	Optimal (>15%)	−4.2 ± 1.4	−17.2 ± 2.0
P5	Low	High	Low (<5%)	Optimal (>15%)	−3.9 ± 1.2	−16.5 ± 1.8
P6	Intermediate	Low	Optimal (>15%)	Low (<5%)	−17.8 ± 2.0	−4.0 ± 1.3
P7	Intermediate	High	Low (<5%)	Intermediate (5–15%)	−4.3 ± 1.5	−8.5 ± 2.2
P8	High	Low	Optimal (>15%)	Low (<5%)	−18.3 ± 2.2	−3.7 ± 1.1
P9	High	Intermediate	Intermediate (5–15%)	Low (<5%)	−10.1 ± 2.3	−4.2 ± 1.4

Response categories: optimal, >15% body weight reduction; intermediate, 5–15%; low, <5%. Each profile: *n* = 10 (5 per arm). ΔBW, percentage change in body weight from baseline to six months; data are mean ± SD.

**Table 3:** Secondary outcomes: changes in waist circumference and HOMA-IR by response category.

Response Category	Drug	*n*	ΔWaist (cm)	ΔHOMA-IR	*p*-Value (Within-Group)
Optimal (>15% BW loss)	Tirzepatide	15	−18.2 ± 3.5	−4.5 ± 1.2	<0.001
Optimal (>15% BW loss)	Semaglutide	10	−16.8 ± 3.1	−4.0 ± 1.0	<0.001
Intermediate (5–15%)	Tirzepatide	10	−9.5 ± 2.8	−2.1 ± 0.8	<0.001
Intermediate (5–15%)	Semaglutide	5	−8.2 ± 2.5	−1.8 ± 0.7	0.002
Low (<5%)	Tirzepatide	20	−3.8 ± 1.5	−0.5 ± 0.4	0.08
Low (<5%)	Semaglutide	30	−3.5 ± 1.3	−0.4 ± 0.3	0.12

Data are mean ± SD. ΔWaist, change in waist circumference from baseline to six months; ΔHOMA-IR, change in HOMA-IR from baseline to six months. *p*-values from paired *t*-tests comparing baseline to six months within each response category. BW, body weight.

## Data Availability

The data presented in this study are available on request from the corresponding author (sandrolavignera@unict.it). The data are not publicly available due to privacy restrictions and ethical considerations related to patient confidentiality.
